# Commentary: Cost-Effectiveness Analysis of Rivaroxaban Plus Aspirin Compared With Aspirin Alone in Patients With Coronary and Peripheral Artery Diseases in Italy

**DOI:** 10.3389/fcvm.2022.916705

**Published:** 2022-06-23

**Authors:** Shalin Rawal, Kamal Sharma, Aditya Shah, Shriya Bavishi, Cleris Christian, Parjanya Bhatt, Ashwati Konat

**Affiliations:** ^1^Intern MBBS, B. J. Medical College and Civil Hospital, Ahmedabad, India; ^2^Department of Cardiology, SAL Hospital, Ahmedabad, India; ^3^Department of Zoology, Biomedical Technology, and Human Genetics, Gujarat University, Ahmedabad, India

**Keywords:** cost-effectiveness, rivaroxaban, dual pathway inhibitor, incremental cost-effectiveness ratio (ICER), Quality-Adjusted Life-Years (QALY)

## Introduction

Rivaroxaban, along with aspirin, is given to reduce cardiovascular risk in patients who have suffered a previous heart attack or stroke. This dual pathway inhibition combination (DPI) is more effective than conventional aspirin alone in preventing recurrent cardiovascular diseases and also causes a significant reduction in mortality, as confirmed by the ATLAS-ACS-2-TIMI-51 studies (1–3). Although rivaroxaban is more expensive, it is considered cost-effective, as it is also assessed by its impact on QALY (Quality-Adjusted Life-Years). The COMPASS trial (4) evaluated the effect of this DPI in clinically stable patients with Coronary artery disease (CAD) and Peripheral Artery Disease (PAD). Based on all these trials, Ferrara et al. shed light on the cost-effectiveness of the combination of low-dose rivaroxaban and aspirin in preventing adverse cardiac events. The study was done in the context of Italy. The various factors considered by the author to calculate the cost-effectiveness include:

- ICER (Incremental Cost-Effective Ratio): defined by the difference in cost between two possible interventions, divided by the difference in the health outcomes or effect.
- QALY: (Quality-Adjusted Life-Years) measures the disease burden, which in simple terms measures both the quality and quantity of life lived.
- Willingness to pay (WTP): Maximum amount of money a typical patient is prepared to spend—set at a threshold value of €40,000 (thrice the gross domestic product per capita of Italy).

A Markov model was used to analyze this data. The cost-effectiveness was calculated as the ICER per QALYs gained, which is the cost to the patient for each additional year of life gained due to the treatment.

## Discussion

The primary goal was to determine the cost-effectiveness of the DPI compared to aspirin alone which was done using statistical analysis. The study subjects who met the criteria for CAD, PAD, or both were derived from the COMPASS study for analysis. CAD patients aged: <65 years with a history of atherosclerosis or at least two additional risk factors like smoking, diabetes mellitus, and glomerular filtration rate: <60 ml/mins were included. The study was the first to classify the population into six categories: CAD or PAD, CAD, PAD, CAD and PAD, CAD with CKD (Chronic Kidney Disease), and CAD with HF (Heart failure). This made the analysis more comprehensive and distinguished it from other studies.

Using the Markov model, patients were classified into multiple mutually exclusive health states expressed by disease parameters. Furthermore, a patient could transit from one state to another. For example, a patient may enter the model in an event-free state and experience any one event: MI (Myocardial infarction), Ischemic Stroke (IS), and intracranial hemorrhage (ICH) or remain event-free. The study of recurrent ischemic events and the impact of post-acute health state on cost-effectiveness is unique to this study.

**Figure 1:**
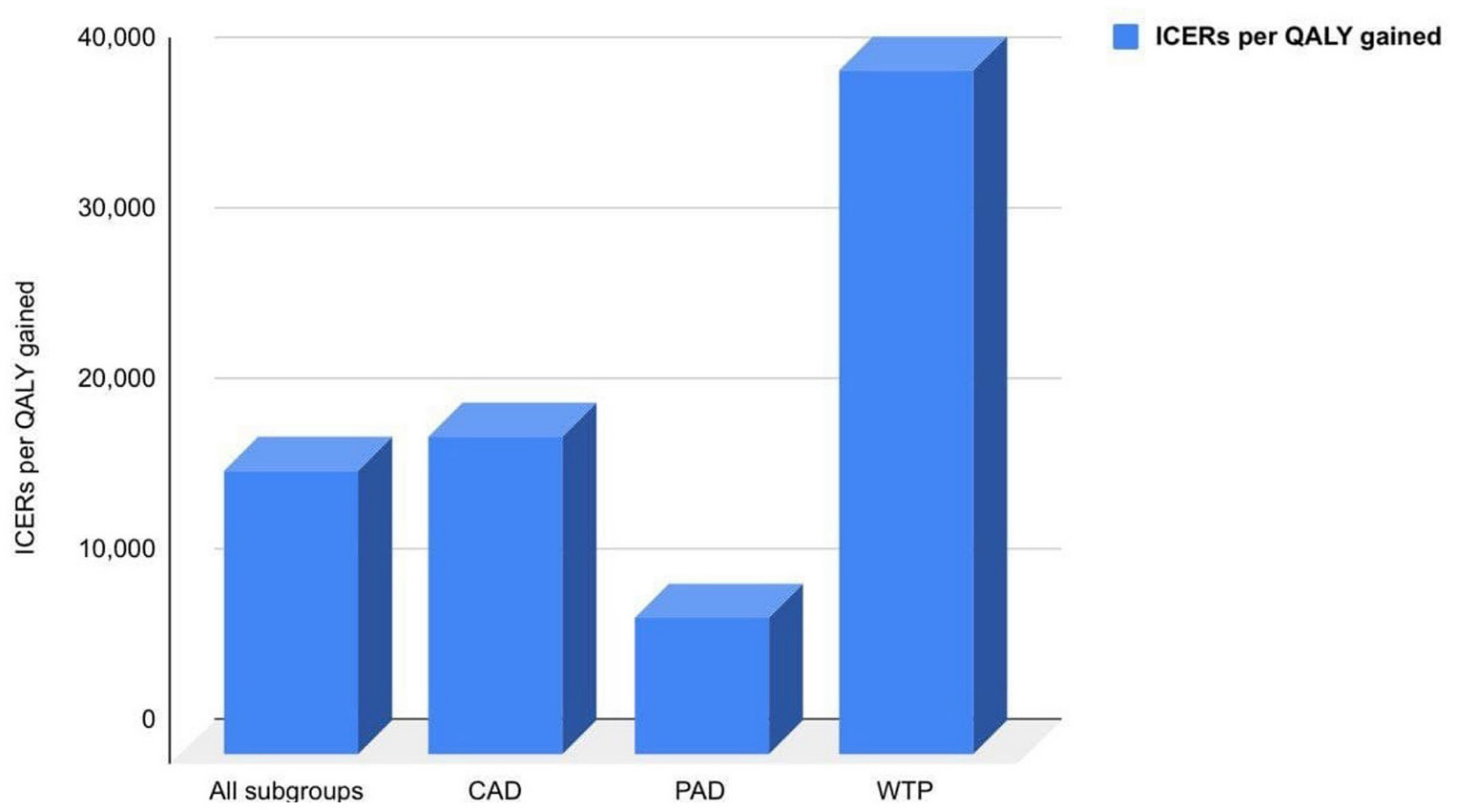
ICERs per QALY gained in different patient subgroups compared to the WTP of the population.

As per the COMPASS trial, DPI markedly improved cardiovascular outcomes than aspirin alone with a respective 240 and 18% relative risk reduction of major adverse cardiac events (MACE), including MI, IS, and all causes of death. The QALY for each treatment and expenditures associated with other healthcare events were obtained for cost-effectiveness analysis. DPI was more effective than aspirin alone, with average QALYs of 9.62 and 9.27 for patients in the population groups of CAD and PAD, respectively. In all populations studied, the ICER for rivaroxaban plus aspirin was €16,522 per QALY gained, based on incremental costs and efficacy, which was significantly less expensive than the WTP threshold of €40,000 per QALY gained. Similarly, in CAD and PAD patients, ICERs were €18,599 and €8,003, respectively, as shown in Figure 1. As the treatment duration increased, ICER dropped by 13% after the first 5 years.

In all subgroups studied, the drug combination was found to be cost-effective, especially in patients with PAD or CAD with HF, which was also seen in multiple analyses globally (5, 6). Furthermore, this cost-effectiveness is maintained across various events, as described earlier. According to the deterministic sensitivity analysis, the main factors affecting the ICER were: Age and treatment efficacy in terms of primary health event rates. The probabilistic sensitivity analysis recalculated the ICERs by varying multiple interplaying factors such as specific scenarios on treatment discontinuation and persistence and confirmed that DPI was cost-effective in nearly all simulations, with 90% of simulations falling below €25,000 in line with QALY gained. Such a comprehensive approach is remarkable and differentiates it from other studies.

The Italian National Tariff was used to assess the prices of adverse outcomes such as intracranial hemorrhage, acute limb ischemia, minor amputations, etc., based on the Diagnosis Related Group (DRG) 2013 tariffs. All medicine prices were collected from the tariffs of the Italian Agency of Medicine.

This research project was wellrounded. However, it had some limitations. Firstly, data was gathered over a relatively short period of 23 months. This information was extrapolated and believed to be constant throughout a person's life. Secondly, the COMPASS population was used in the analysis. The likelihood and danger of health problems in real-world populations are higher; therefore, the DPI maybe even more cost-effective. This difficulty could be solved by extending the study's duration, and using a larger sample size representative of real-world health event risks. Furthermore, DRG tariffs used for cost modeling can be variable due to the lack of data from some hospital-level cost studies. Additionally, the author may have included diabetic subgroups in the CAD/PAD population and included the comparison of clopidogrel and rivaroxaban, both of which are mentioned in other studies (7). The article scored 94/100 in the Quality of Health Economic Studies (QHES) checklist (8) and also followed the Consolidated Health Economic Evaluation Reporting Standards (CHEERS) checklist (9).

## Conclusion

This study was a side-by-side assessment of two different therapy options to prevent adverse cardiac effects. A cost-effectiveness analysis is essential to rationalize decision-making in resource constraints, especially in the pandemic era. In all subgroups, especially the high-risk groups with increased comorbidities, DPI shows higher efficacy and cost-effectiveness. Extrapolating this model in various countries can provide a complete picture of the superiority of DPI over aspirin.

## Author Contributions

All authors listed have made a substantial, direct, and intellectual contribution to the work and approved it for publication.

## Conflict of Interest

The authors declare that the research was conducted in the absence of any commercial or financial relationships that could be construed as a potential conflict of interest.

## Publisher's Note

All claims expressed in this article are solely those of the authors and do not necessarily represent those of their affiliated organizations, or those of the publisher, the editors and the reviewers. Any product that may be evaluated in this article, or claim that may be made by its manufacturer, is not guaranteed or endorsed by the publisher.
