# Supplementary material for: Commentary: Cost-Effectiveness Analysis of Rivaroxaban Plus Aspirin Compared With Aspirin Alone in Patients With Coronary and Peripheral Artery Diseases in Italy
Source: Front Cardiovasc Med. 2022 Jun 23;9:916705. doi: 10.3389/fcvm.2022.916705 (PMC9260166; doi:10.3389/fcvm.2022.916705)
Supplement: Supplementary file 1 [file Table_1.pdf]

The Quality of Health Economic Studies (QHES) instrument

Taken from: Ofman JJ, Sullivan SD, Neumann PJ, Chiou CF, Henning JM, Wade SW, Hay JW. Examining the value and quality of health economic analyses: implications of utilizing the QHES. Journal of Managed Care and Pharmacy 2003; 9(1):53-61.

No	Questions	Points	Yes	No
1.	Was the study objective presented in a clear, specific, and measurable manner?	7	✓	
2.	Were the perspective of the analysis (societal, third-party payer, etc.) and reasons for its selection stated?	4	✓	
3.	Were variable estimates used in the analysis from the best available source (i.e., randomized control trial - best, expert opinion - worst)?	8	✓	
4.	If estimates came from a subgroup analysis, were the groups pre-specified at the beginning of the study?	1	✓	
5.	Was uncertainty handled by (1) statistical analysis to address random events, (2) sensitivity analysis to cover a range of assumptions?	9	✓	
6.	Was incremental analysis performed between alternatives for resources and costs?	6		✓
7.	Was the methodology for data abstraction (including the value of health states and other benefits) stated?	5	✓	
8.	Did the analytic horizon allow time for all relevant and important outcomes? Were benefits and costs that went beyond 1 year discounted (3% to 5%) and justification given for the discount rate?	7	✓	
9.	Was the measurement of costs appropriate and the methodology for the estimation of quantities and unit costs clearly described?	8	✓	
10.	Were the primary outcome measure(s) for the economic evaluation clearly stated and did they include the major short-term, long-term, and negative outcomes?	6	✓	
11.	Were the health outcomes measures/scales valid and reliable? If previously tested valid and reliable measures were not available, was justification given for the measures/scales used?	7	✓	
12.	Were the economic model (including structure), study methods and analysis, and the components of the numerator and denominator displayed in a clear, transparent manner?	8	✓	
13.	Were the choice of economic model, main assumptions, and limitations of the study stated and justified?	7	✓	
14.	Did the author(s) explicitly discuss direction and magnitude of potential biases?	6		✓
15.	Were the conclusions/recommendations of the study justified and based on the study results?	8	✓	
16.	Was there a statement disclosing the source of funding for the study?	3	✓	
	**TOTAL POINTS**	**100**	88	
